# Identity, trust, and the experiences of refugees during a COVID-19 lockdown

**DOI:** 10.1371/journal.pone.0271977

**Published:** 2022-08-01

**Authors:** Melati Nungsari, Hui Yin Chuah, Sam Flanders

**Affiliations:** 1 Asia School of Business, Kuala Lumpur, Malaysia; 2 MIT Sloan School of Management, Cambridge, MA, United States of America; Newcastle University, UNITED KINGDOM

## Abstract

This paper examines the experiences of refugees in a developing country during its first COVID-19 lockdown by utilizing a two-stage qualitative data analysis of 39 interviews with refugees and asylum-seekers. We find that their experiences during the lockdown are shaped by identity, trauma and help from external parties–such as community leaders and local non-governmental organizations (NGOs). Experiences during the pandemic in turn moderate the relationship between policy changes and trust in domestic authority figures, which consequently affects attitudes towards and compliance with public health measures put in place to contain the pandemic. We then explore the role of identity in refugees’ pandemic experiences by comparing the differences between two refugee groups (Syrians and Rohingyas), validating them by utilizing comparative thematic analysis. Finally, the paper presents policy implications for crisis response in developing countries by suggesting improvements that can be made on the ground regarding the delivery of aid and assistance to vulnerable groups.

## Introduction

The world is still reeling from the spread and severity of the COVID-19 pandemic since it was declared a global health concern in March 2020 by the World Health Organization [1]. Its impacts are particularly salient for developing countries such as Malaysia, which may not have adequate capacity particularly in terms of resources in addressing the public health crisis. Malaysia recorded its first COVID-19 case on January 25^th^, 2020 –an imported case that arrived in the state of Johor via Singapore [2]. After a large religious gathering in its capital city, Kuala Lumpur, which then spurred the spread of the virus across Southeast Asia, the country was placed under a “Movement Control Order” (MCO)–a strict, nationwide lockdown that involved the general prohibition of mass movements and gatherings, the closing of national borders to foreigners, prohibitions for Malaysians against leaving the country and closures of all schools, officers, universities, public and private premises, among others [3]. During this period, the government conducted mass testing operations around the country, initially conducted regardless of one’s immigration and documentation status: individuals exhibiting symptoms were told to come forth or comply with contact tracing activities without any fear of repercussion or harm. However, in May, authorities conducted at least four immigration raids and arrested more than 2,000 undocumented persons, including asylum-seekers, refugees, and children [4]. Additional policies were also enacted, including the restrictions against foreigners praying in mosques [5].

Malaysia has a long history of accommodating and hosting refugees in its post-independence period, dating back to the 1970s with the arrival of the Vietnamese “boat people” [6]. It hosts at least 178,000 refugees and asylum-seekers from 50 countries, including the largest number of refugees from Myanmar after Bangladesh, most of whom are Rohingyas [7, 8]. Despite this, Malaysia has not signed the 1951 United Nations Convention Relating to the Status of Refugees or its 1967 Protocol, thus implying that refugees in Malaysia do not have any legal status [9], with the official stance being that Malaysia accepts and hosts refugees *only* until they can be permanently resettled in other countries, such as the United States and Europe [10]. This official stance as a transit country has resulted in extremely precarious living and working conditions for refugees in Malaysia, as they cannot formally access public education and affordable healthcare services, and also lack the right to work legally [11–14]. These factors have been further exacerbated by the COVID-19 pandemic as the refugee populations continue to be sidelined despite the formulation of new policy responses, posing a unique set of challenges for this population [15]. For example, during immigration raids, refugees were often lumped together with undocumented persons and migrants who were illegally residing in the country, despite the fact that they were formally registered with the United Nations High Commissioner for Refugees (UNHCR) and possessed relevant identification cards to demonstrate this fact [16].

The literature surrounding issues related to the pandemic is rapidly expanding every day. We know, for example, that in the United States, low-income students are disproportionately affected by school shutdowns [17], individual choices tied to fears of infection seem to be the reason for the collapse in economic activity rather than the declaration of legal shutdown orders [18] and that the structure of social networks plays a very important role in determining the geographic spread of the disease [19]. The data on the impacts of the pandemic on emerging economies, however, are sparse. Furthermore, relevant studies tend to focus on the general population with little to no regard for resident vulnerable populations such as refugees and displaced persons. Although some attention has been given to understanding and measuring the health risks of refugees in camps and urban slums [20–23] as well as migrant workers [24], there have not been very many attempts to address the experiences of refugees within a country–and thus a lack of understanding of the socioeconomic impacts of the pandemic on their lives, or how different interventions can improve their lived experiences and compliance with public health measures.

Thus, this study aims to answer two main questions: 1) how have pandemic-related lockdowns affected refugees in Malaysia, a transit country; and 2) what are the mechanisms by which trust affects compliance with and attitudes towards public health measures, and how does identity play a role in this process?

We answer these questions in two steps. First, we use qualitative data obtained from 20 refugee community leaders from eight distinct groups to inductively build a theoretical framework to understand the refugee experience during the first MCO. Throughout its duration, Malaysia underwent several nationwide policy changes–these included free mass testing for all (including refugees and undocumented migrants) as well as increased numbers of immigration raids. Our conceptual model posits that one’s experiences can moderate the relationship between policy changes and trust in local authority figures, which then affects attitudes towards and compliance with public health instructions regarding transmission suppression efforts. In our conceptual model, we also find that refugee experiences are influenced by multiple factors including identity, trauma, refugee community leaders and local NGOs. Among these, identity plays a major role. To answer the second research question, we further explore the relationship between identity, trust and experiences by conducting comparisons between two distinct refugee groups–the Rohingyas and Syrians.

This paper has a broader relevance for other developing countries, since 85% of refugees worldwide are hosted in “developing regions”, including Malaysia [25]. Furthermore, the level of compliance with public health recommendations amongst vulnerable, and often hidden, groups is very relevant moving forward, particularly in an interconnected world rife with infectious diseases. A deeper understanding of the experiences of refugees needs to be taken into consideration in developing resilient preparation and response mechanisms. Section 2 presents the literature review, Section 3 presents the methodology and data, Section 4 presents the findings, Section 5 presents a policy discussion and Section 6 concludes the paper.

## Literature review

Trust has been well explored in different contexts, including public health and social science [26–28]. Many studies have examined trust in healthcare systems and vaccine hesitancy during the Ebola outbreak [29–31]. The literature on the relationship between trust and outcomes such as trade or economic development has shown that trust improves citizen involvement and governmental performance [32–35], tax compliance [35, 36] and decisions to report crimes [37]. Trust or mistrust is also widely discussed as one of the factors shaping the experiences of refugees during exile or after resettlement [38–43]. However, the issue of trust rarely takes centre space in refugee studies [38].

One of the key reasons is the challenge in defining the abstract and multidimensional concept of trust. While its definition varies, scholars all point to trust involving a dependency on another party or parties. For instance, Rothstein defines trust as “a bet on the behavior of others” [44], while according to Mayer, Davis, and Schoorman, trust is the “willingness of a party to be vulnerable to the actions of another party based on the expectation that the other will perform a particular action important to the trustor, irrespective of the ability to monitor or control that other party”[45]. We utilize the definition by McKnight, Cummings, and Chervany [46], where trust is “a combination of trusting beliefs, defined as the belief that another is benevolent, competent, honest, or predictable, in a given situation, and trusting intentions, meaning one’s willingness to depend on another in a situation”.

The concept of trust is of great significance in refugee studies. In investigating the role of trust in the experiences of refugees, Lyytinen (2017) and Hynes (2009) have divided trust into social trust and institutional trust, in which social trust refers to sense of trust among refugees and other people they interact with, while institutional trust refers to trust in institutions providing protection or services to refugees [42, 47]. Social trust can be further sub-divided into particularized and generalized social trust. The former refers to trust in people that you know, while the latter refers to trust in strangers [42]. Trust is a dynamic construct that is shaped and being reshaped over time and throughout the journey of refugees [42, 48]. It is also context-dependent [38, 49]. Hynes analysed how trust among refugees change in different contexts: from the period of threat in their country origin, to forcibly displaced and seeking exile and finally resettled [47]. Trust also shapes and is shaped by the intersection of social identities such as gender, socioeconomic status, and ethnicity [42, 50]. According to Smith, “race is the most important determinant of trust”[51].

In crisis, the role of the government in communications as well as the provision of accessible and accurate information becomes more crucial than ever. With the COVID-19 pandemic came a variety of policies designed to stop or mitigate its spread, such as stay-at-home orders, complete lockdowns, social distancing orders as well as mass screening and testing. While no one has been spared from the crisis, the pandemic and its related containment measures have disproportionately harmed the most marginalized populations including minorities, women, people of low socioeconomic status, undocumented migrants and refugees, and some of these categories intersect [52, 53]. The effective implementation of relevant policy responses relies heavily on public compliance, among other factors. A variety of factors play a role in public compliance, with the main one being trust.

Social trust plays a crucial role in public health crises given the serious disruptions to the existing social system and heightened uncertainties [54]. Degraded social trust and cohesion in a society does not only have significant socioeconomic consequences but may also cause substantial negative impacts when compliance is required for collective survival [55]. Bargain and Aminjonov also found a positive relationship between community attachment (which is mainly manifested by social trust) and higher outcomes of preventative behavior. Mobility restrictions and social distancing are among the first primary policy responses imposed against COVID-19. In Switzerland, Deopa and Forunato found a strong association between levels of trust and compliance with social distancing measures [56]. The study illustrated that higher generalized social trust leads to lower reductions in individual mobility during the pandemic. Also, a study in the United States suggests that there is stronger compliance with social distancing orders when individuals’ social trust is higher [57].

In the context of COVID-19, it is important to note that blindly replicating lockdown measures in high income countries may cause harmful effects and spark social unrest, particularly in communities with high poverty rates and low institutional trust [58]. A study of the influence of social media on police legitimacy found that people are more likely to comply with governments when they trust the government and view it as legitimate [59]. Legitimacy does not matter only to citizens, but also refugees and asylum-seekers, and thus, their trust in the government plays a role in ensuring their compliance with public policies [60]. The same study also found that the refugee perceptions of the Turkish government are shaped by domestic ethnic conflict and political circumstances as well as their host community’s (Turkish citizens) own perceptions of the government. Other factors such as cultural and language barriers as well as the host society’s approach to refugees also have an effect on the ties and interactions between refugees and host governments [61, 62], which in turn may influence the institutional trust and hence the impacts of COVID-19 and lockdown measures on the refugee population.

## Methodology

### Data collection

**Fig 1:**
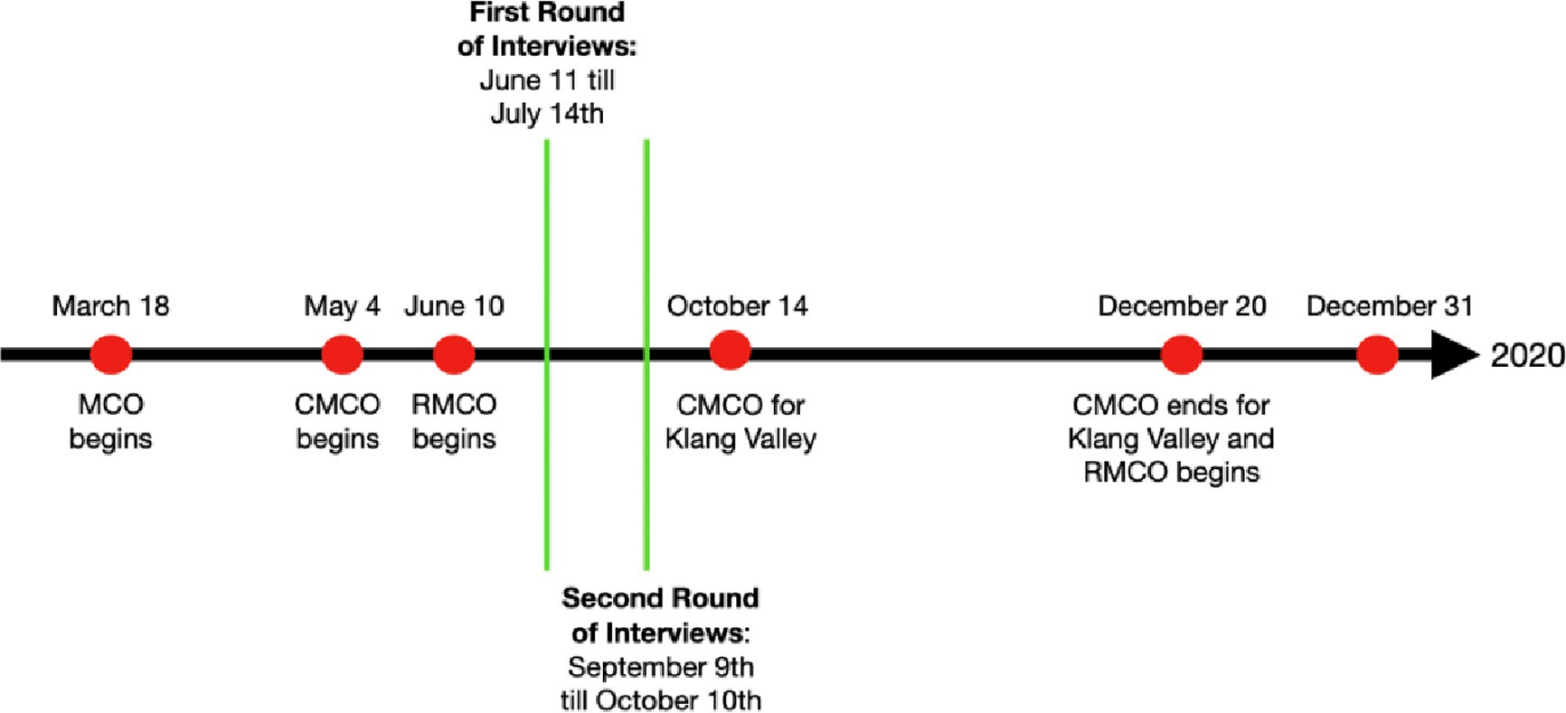
Timeline of interviews and the lockdowns (i.e. MCO, CMCO and RMCO).

The Malaysian government enforced a strict nationwide MCO from March 18^th^ to May 4^th^, 2020, following a spike in COVID-19 cases. Schools, workplaces and most non-essential economic sectors were closed, public transportation was halted, all gatherings outside the household were banned and borders were closed to all non-citizens. Subsequently, the country started to reopen by loosening restrictions in phases, starting with the “Conditional MCO” (CMCO), which went into effect from May 5^th^ until June 10^th^. More restrictions were loosened between June 11^th^ and mid-October under the “Recovery MCO” (RMCO). At that point, Malaysia started recording large spikes in recorded cases due to spillovers from political campaigning and a state election in the heavily hit Bornean state of Sabah, causing a return to CMCO conditions, with some states, including the Klang Valley area (the metropolitan area that includes Kuala Lumpur), placed under stricter restrictions starting October 14^th^, before reverting to the RMCO in December. The MCO was reimposed three times in 2021. The timeline of the data collection is depicted in Fig 1.

Approval was sought and obtained from the Asia School of Business’s Institutional Review Board (IRB) prior to the study commencing. In line with the protocol that was approved, and due to the fact that data was collected during a pandemic with physical distancing measures in place, verbal consent was obtained from all participants who were interviewed. This was done after the researcher had assessed the capacity of the individual to provide consent through casual, non-recorded talk prior to the start of the (recorded) interview. Participants were referred to us from the following agencies and organizations: the United Nations High Commissioners of Refugees (UNHCR), the Rohingya Women Development Network (RWDN), the Mon Refugees’ Organization, the Kachin Refugee Committee, the Malaysia Karen Organization, the Somali Refugee Community of Malaysia, and the Malaysian Social Research Institute (MSRI). Participants were not held in refugee camps during the interviews as this is not a current practice in Malaysia. The first two authors of this paper conducted the interviews either together or individually with the participants. All participants either spoke Bahasa Malaysia or English and participants were given the agency to choose which language would be used for the interview.

The first round of interviews was conducted from June 11^th^ to July 14^th^ 2020 involving 20 refugees (six women and 14 men) from Afghanistan, Myanmar, Somalia and Syria. The respondents from Myanmar can be further broken down into five distinct refugee groups according to the data categories provided by the UNHCR: namely the Kachin, Karen, Mizo, Mon and Rohingya. This granular breakdown could not be done for refugees from other countries since the size of their refugee populations is too small. The respondents were selected by asking multiple local NGOs for the contact details of “refugee community leaders” in various locations around the country. This first round of interviews was based on semi-structured questions that focused on five general topics–communication of information, social lives, security, economic factors and health. The data was then analyzed and used to build a theoretical framework and process map of a generalized refugee’s experience during the pandemic, as described below.

The second round of interviews was conducted with two separate groups of refugees who identified themselves as Rohingya and Syrian respectively, and the results were analyzed in comparison to each other. Individuals from the first round of interviews were not interviewed again. Rohingyas were chosen as the base group for comparison because they are the majority refugee group in Malaysia, constituting approximately 57% of refugees [8]. During the pandemic, Rohingyas were also singled out among other refugee groups and targeted in the media, on the ground by members of the public as well as systematically targeted by the government. It was reported in April that “the country’s sizeable population of Rohingya Muslim refugees are facing a torrent of xenophobia … petitions calling for the refugees to be deported–a violation of international law–have flooded online spaces, while there has been an uptick in vitriolic anti-immigrant comments on social media” [63]. Hundreds of undocumented migrants, including Rohingya refugees, were detained as part of a widespread immigration campaign disguised as a policy response to contain COVID-19 since May [20]. In June, Malaysian authorities also detained 269 Rohingya refugees who arrived on a damaged boat in Langkawi, an island off the northwestern West Malaysian coast [64]. This contrasted sharply with the sympathetic attitude shown by Malaysians, particularly Muslims, towards Rohingya refugees prior to the pandemic, where public protests against the genocide of Rohingya people in Myanmar were led by former Prime Minister Najib Razak in 2016, despite the core principle of non-interference adopted by the Association of Southeast Asian Nations (ASEAN), of which both Malaysia and Myanmar are member states [65]. While the Syrian refugees are not comparable to the Rohingya in terms of their population size, general sentiments among Malaysians towards them are similar. Donations to Syrian refugees in Lebanon and Turkey were mobilized among the public [66] and the government even offered to host 3,000 Syrian refugees who had to flee the Syrian war [67]. Culturally, Syrians bear some similarities to Rohingyas: the majority are Sunni Muslim (as are most citizens in Malaysia), and they speak and read Arabic, the language of the Quran [16].

Sampling for the second round of interviews was done through snowballing, based on information from the refugee leader of the same group in the first round. Semi-structured interviews were held from September 9^th^ until October 10^th^, focusing on the respondents’ experiences from the first MCO until the present, and their experience interacting with government authorities in Malaysia. We ran a thematic analysis in parallel with the data collection. The snowballing and recruitment of more respondents ended as soon we reached a saturation in themes–i.e. the point where an additional transcript did not add to the set of already existing themes extracted from prior respondents [68]. In total, 11 Rohingyas (10 men and one woman) and eight Syrians (two women and six men) were interviewed.

### Data analysis

We first utilized our qualitative data from the first round of interviews to inductively build a model of the refugee experience starting from the MCO by utilizing grounded theory [69]. We built our theory by systematically collecting and analyzing data in order to understand how our respondents interpreted their realities, and how they defined different agents in their lives. Following the suggestions by Suddaby [70], we analyzed these interviews by utilizing two main concepts: 1) constant comparison (in which we collect data and analyze them in parallel); and 2) theoretical sampling (in which decisions surrounding what issues to probe in subsequent interviews are determined by the theory that is being constructed).

The second set of interviews was collected to explore a particular piece of the emergent model–specifically, the role of identity in shaping trust and experiences of the refugees. To do this, we interviewed two separate groups of individuals who identified as Rohingya and Syrian respectively by utilizing identical semi-structured questions. There is no overlap in terms of the interviewees. We first coded the interview transcripts of each group separately, conducting a thorough thematic analysis [71]. We then compared and contrasted the set of themes that emerged from each of the groups by noting differences as well as commonalities.

## Findings

### A process model of a refugee’s experience during the COVID-19 lockdown

**Fig 2:**
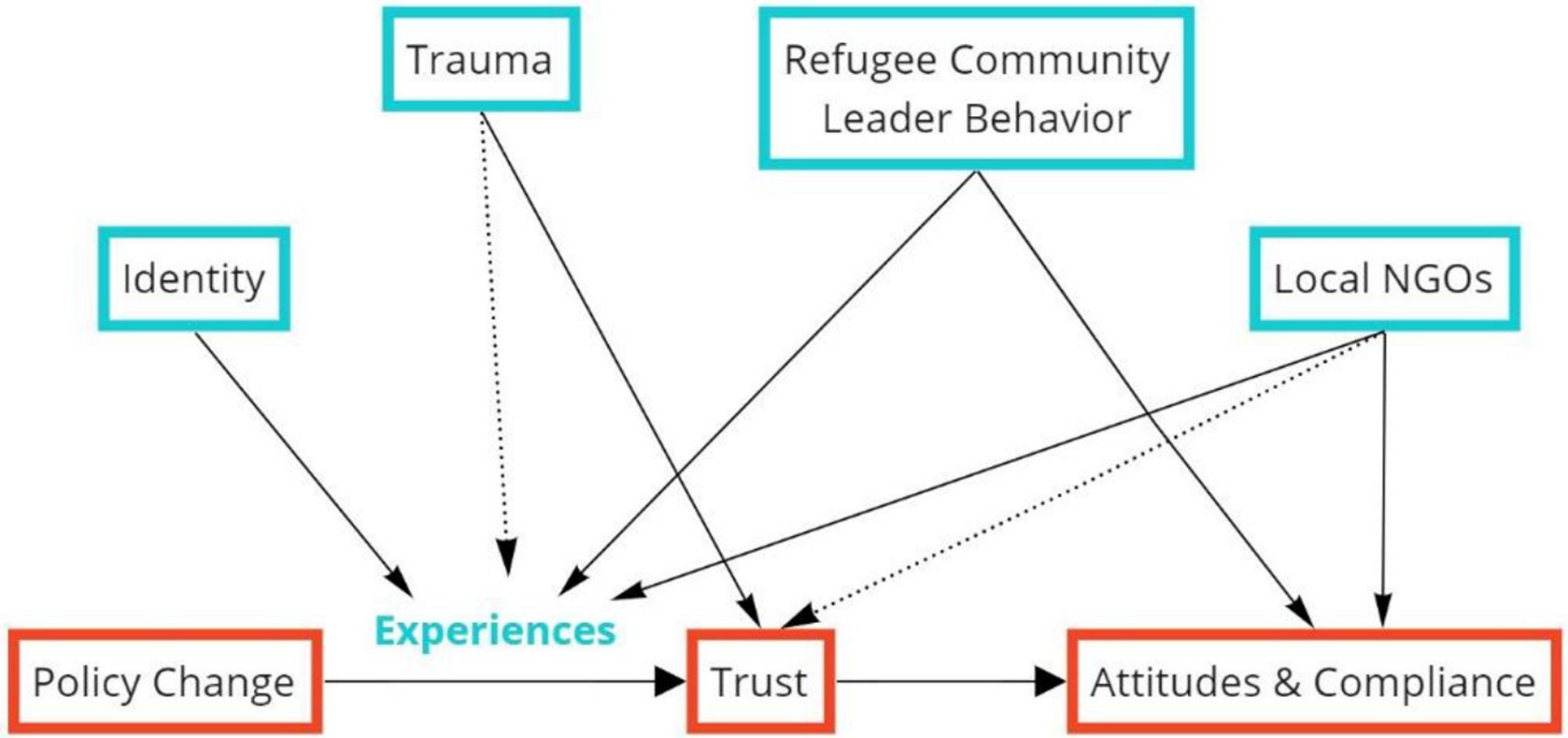
A process model of a refugee’s experience starting from the implementation of the MCO. The arrows represent associations between two factors, the solid lines represent strong association, and dotted lines represent modest association.

With reference to Sutton [72] which utilized qualitative evidence and process model in the study of organizational death, we inductively built a theoretical framework to illustrate the process flow of a refugee’s experience during the first COVID-19 lockdown as well as the factors affecting the refugee’s experience in the process flow. By applying grounded theory [69], we employed a systematic method of constant comparisons between the qualitative data collected and the model that was built. Although we did not work in a strictly linear fashion, the general steps were as follows: first, all interviews were transcribed verbatim, according to the language that they were conducted in, before being translated into English if the transcript was in a non-English language. Then we conducted a detailed thematic content analysis as in [71] to extract the emergent themes from the interview data, with each topic-level being the unit of analysis (i.e. each transcript was broken down into different pieces or “excerpts” according to the different topics discussed). The full list of themes is displayed in Table 1. We then consulted the data again to recontextualize these themes, and the model was constructed according to most of the main variables and agents that emerged: experiences, local NGOs, refugee community leaders, trauma, attitude, policy changes, trust and identity. We further conducted a cross-site display of evidence from the data obtained for our 20 respondents to validate our process model, as in [73, 74] (see Table 2). It is crucial to note that “despite this systematic effort, a one-to-one match between model and data was not observed, not was it expected” [72], and that the process model that was built here, although it explains the data well, does not (and cannot) fit all qualitative evidence perfectly [75]. The resulting model is displayed in Fig 2. This model involved repeated triangulation between all researchers involved in the study and was validated with respondents prior to the second round of interviews.

**Table 1:** Themes obtained from the thematic content analysis of first-round interview data.

**SECURITY**
• Experiences in enhanced strict lockdown areas (EXP, TA) • Increased need and important for UNHCR registration (EXP, A) • Experiences with detention centres and jails (EXP, TA) • Interactions with government policies (EXP, TA, TU) • Police and immigration raids (EXP, TA, TU, P) • Deportation (EXP, TA, TU) • Placing blame of Rohingya (EXP, ID) • Fear of going out (A) • Reliance on community-based organizations and local NGOs (EXP, N) • Erosion of trust towards authorities (TU) • Detention of minors (EXP, TA, TU) • Roadblocks as a source of fear and arrests (EXP, T) • Negative interactions with Malaysians (EXT, T, ID) • Repatriation as a solution to negative experiences in Malaysia (AT) • Resettlement as a solution to negative experiences in Malaysia (A, T)
**SOCIAL**
• Resilience (A) • Fear, shame, and perseverance (A) • Appreciation to Malaysians (A) • Frustration towards government policies (EXP, P) • Acceptance and understanding of government policies (EXP, P) • Advocacy on the right to work (A) • Targeted hate towards Rohingya (EXP, ID) • Discrimination and hate towards refugees (EXP, TA, TU, ID) • Discrimination and discontent amongst different refugee groups (EXP, ID) • Assistance from local NGOs (EXP, N) • Collaborations between local NGOs and CBOs (EXP, N, L) • Refugee led community-based initiatives (EXP, L) • Aid from private organisations and individuals (EXP, N, L) • Support from social networks (EXP, N, L) • Negative impacts on kids (EXP, TA) • Increased challenges for women and single mothers (EXT, TA) • Increased SGBV cases and difficulties handling them within the community (EXP, A, TA) • Changes in family dynamics with mothers earning money due to fathers losing jobs (EXP, A) • Disrupted access to community-based education due to lockdown (EXP, TA, N, L) • No access to education in some states (EXP, TA)
**COMMUNICATION**
• Illiteracy (EXP) • Poor connectivity (EXP) • Community leaders and refugee CBOs (EXP, N, L) • Physical outreach (EXP, N, L) • Social networks (EXP, N, L) • Social media (EXP, N, L) • Malaysian local NGOs (EXP, N, L)
**HEALTH**
• Free from COVID-19 (EXP) • Suspected cases and symptoms (EXP, A, P) • Fear of contracting the virus (EXP, A) • Unverified rumours (EXP, A) • Awareness of COVID-19 symptoms (EXP, AP) • COVID-19 positive cases (EXP, A, P) • Required COVID-19 testing for work (EXP, A, P) • Cooperation with contact tracing (EXP, A, P) • Scepticism with contact tracing (EXP, A, P) • Lack of trust in government (EXP, TU) • Financial issues (EXP, TA) • Lack of documentation (EXP, TA) • Fear of going to the hospital (EXP, A) • Security concerns (A) • Restricted access to healthcare during the lockdown (EXP, TA) • Mental health issues for kids (EXP, TA, A) • Mental health issues for women (EXP, TA, A) • Mental health issues for community leaders (EXP, TA, A) • Mental health issues for community members (EXP, TA, A) • Local NGO clinics as alternatives to public healthcare (EXP, N, P) • Private providers as alternatives to public healthcare (EXP, N, P)
**ECONOMIC**
• Job loss from the economic slowdown and lockdown (EXP, P) • Job loss due to changes in government policy (EXP, P) • Reduction in pay from some who remain employed (EXP, P) • Women finding work to make up for decrease (EXP, P, A) • Reliance on savings (EXP, A) • Difficulties fulfilling basic needs (EXP, P) • Evictions and difficulties paying rent (EXP, P) • Dependence on assistance and handouts (EXP, N, P) • Targeted labour discrimination towards Rohingya (EXP, ID) • Barriers to starting informal business to make ends meet (EXP, A) • Financial and capacity constraints on local NGOs’ a distribution (EXP, NP, P) • Fewer job opportunities after the lockdown (EXP) • Optimism about job opportunities (EXP, A) • Uncertainty about job prospects (EXP, A)

*** “EXP” denotes “Experience”, “N” denotes NGO, “L” denotes refugee community leader, “TA” denotes trauma, “A” denotes attitude, “P” denotes policy change, “TU” denotes trust, and “ID” denotes identity.

**Table 2:** Cross-site display of evidence for the process model. Bolded upper-case letters suggest strong evidence of the particular theme from the first round of interviews, lower-case letters suggest modest evidence of the particular theme from the first round of interviews, and “-” indicates no evidence.

Interviewee	Policy change (P)	Identity (ID)	Trauma (TA)	Community leader (L)	NGO (N)	Experience (EXP)	Trust (TU)	Attitude (A)
Afghan	**P**	-	ta	**L**	**N**	**EXP**	**TU**	**A**
Karen	**P**	id	**TA**	l	**N**	**EXP**	**TU**	**A**
Kachin	**P**	-	**TA**	l	**N**	**EXP**	tu	**A**
Mizo	p	**ID**	ta	l	**N**	**EXP**	**TU**	**A**
Mon	**P**	-	**TA**	**L**	**N**	**EXP**	**TU**	**A**
Rohingya 1	p	**ID**	**TA**	**L**	**N**	**EXP**	tu	a
Rohingya 2	**P**	-	**TA**	-	**N**	**EXP**	**TU**	**A**
Rohingya 3	**P**	**ID**	-	l	n	**EXP**	**-**	**A**
Rohingya 4	p	**ID**	ta	l	n	**EXP**	tu	a
Rohingya 5	**P**	**ID**	**TA**	**L**	n	**EXP**	**TU**	**A**
Rohingya 6	p	-	-	l	**N**	**EXP**	tu	**A**
Rohingya 7	p	id	-	l	**N**	**EXP**	**TU**	**A**
Rohingya 8	**P**	**ID**	-	**L**	**N**	**EXP**	**TU**	**A**
Rohingya 9	p	**ID**	-	l	n	**EXP**	-	**A**
Rohingya 10	p	**ID**	ta	**L**	**N**	**EXP**	**TU**	**A**
Rohingya 11	**P**	**ID**	-	**L**	**N**	**EXP**	**TU**	**A**
Rohingya 12	**P**	**ID**	-	**L**	**N**	**EXP**	**TU**	**A**
Somali 1	p	**ID**	**TA**	**L**	**N**	**EXP**	tu	**A**
Somali 2	**P**	-	-	**L**	n	**EXP**	tu	**A**
Syrian	p	id	ta	-	**N**	**EXP**	**TU**	a

Due to the pandemic, the Malaysian government enacted many policy changes, most of which were unanticipated, that directly affected a refugee’s life. Some examples that were highlighted by respondents included employers who were suddenly no longer allowed to hire refugees (refugees and asylum seekers are treated as undocumented migrants and have no legal rights to work. Nonetheless, many managed to find informal odd jobs prior to the pandemic), house owners who were warned against renting their property to undocumented migrants, the banning of all non-citizens from mosques and restrictive “extra” lockdowns in Enhanced MCO (EMCO) areas which had seen large spikes in cases (incidentally, this also corresponded to areas where many refugees lived), which involved mass testing and sudden pivots from amnesty to raids and detentions of undocumented persons. We found that both positive and negative experiences mediated the relationship between the policy changes and the level of institutional trust a refugee had in the authorities (e.g. public healthcare workers tasked with obtaining samples for mass testing, police, immigration officers, politicians). Trust then directly affects refugees’ attitudes during the pandemic and their compliance with public health measures, such as seeking medical help when exhibiting COVID-19 symptoms and revealing close contacts’ personal information, such as their locations and phone numbers for the purposes of contact tracing. All quotes presented in this subsection are from the first round of interviews.

I heard during MCO, they shared their address, their information with NGO … for the corona test. And unfortunately, immigration went there and they arrested them and they are scared. And, of course … it’s not easy to trust if they give the (information) to others.

We also found a number of main variables that directly (and indirectly) affected refugee experiences, such as identity–the Rohingya were both explicitly targeted by certain policies, as well as by other ethnic refugee groups. One respondent also mentioned that “looking Asian” in order to fit in mattered.

A lot of people suffering, lost their jobs and a lot of Rohingya especially who has also UN card. They have been arrested and sent to the prison and camp … And a lot of things happening, some of the house owners they are asking the Rohingya families to leave the house even though they continue giving the house rent … So, I told you, our lives depend on you and the government and everyone. But I don’t why (campaign against the Rohingya) suddenly become very big … A lot of the online petitions were signed by the Malaysians.

Experiences were also indirectly affected by past or ongoing trauma. In EMCO areas, the military and police put up barbed wire around particular buildings to enforce heightened lockdown conditions–a detail that was brought up by multiple respondents, with some indicating that it reminded them of past violence. Some even compared the ongoing xenophobia in Malaysia to suffering in their country of origin and yearned for repatriation.

I think (the) psychological impact during MCO and after is really deep. Because they think that we’d rather die back in Myanmar or back in my country instead of suffering. They are all very worried, especially for community cardholders. They run away from the country because of the persecution. But then because of the insecurity and the fear that they have is so big that they think that, “oh, we try to find a way and go back (home) … we’d rather die with our family even though we know that the risk is there.” They know that but they want to choose the way because they are scared that they will be detained and locked in the detention center for a long time.

Past trauma also directly impacted trust. For example, respondents signaled that they no longer trusted the authorities as they related the story of refugees residing in EMCO areas who initially complied with orders, only to be detained and deported. Similarly, respondents who had heard of community members being detained became wary of the police themselves. One respondent spoke to us about individuals in their social networks who were tested by authorities during the lockdown and arrested immediately after their two-week quarantine:

Because during lockdown I was informed (that I was) in EMCO area, they are going house to house to do the check-up. Regardless of ethnicity and religion. So, if you are doing check-up, you should wait at least until 14 days after your check-up done to prove that these people don’t have symptoms and they are OK. (Then only would they be) safe enough to be arrested. Even though arrest is not the final solution. But for me I feel like government purposely (conducts these) arrests and detention … before the 14 days test period. Just because they want to show the migrants and refugees are the reason behind COVID.

Ongoing trauma was also mentioned by the interviewees–particularly mental health anguish on the part of children, women, themselves and local contacts who were distributing aid. The lockdown measures also put a huge strain on some households, which contributed to more conflict and even domestic abuse in some families. Despite acknowledgement of these issues, there is little that can be done by the community leaders due to resource constraints, since urgent basic needs, particularly food and shelter, are being prioritized at the moment.

On school-aged children:

So the school(s) shut down, they are at home, not doing anything and looking at the parents and the family member(s). Raids happening … I feel that the damage has already been made … It’s not only to the father and mother, it also goes to the children and this is gonna continue. The children are going to carry this experience of raids and all for their future … I’m more worried on their well-being in the future. Because we know … (a) traumatic experience is not something that we will forget. And for me I don’t know how the children are coping.

On pregnant refugees and domestic abuse:

So we notice two things. One is the pregnant woman things you know. They are having psychological and mental … struggle(s). And delivering baby in government hospital or private. That’s one part. The second part we notice domestic violence cases are coming up you know. Because like (if a) husband lost their job, how to feed the children(?) Family arguing become like more during MCO. And some, they don’t have an outlet for all their stress and problems so they throw everything on the wife. That’s when they start … fighting and some cases like physical abuse involved and stuff. These are the consequences and this is the impact (of) the virus and the lockdown and MCO.

The behavior of refugee community leaders also directly impacted their experiences, and often affected the attitudes and compliance of both themselves and the communities they led. Some were generally more optimistic about their situation and ability to provide for their communities, which made for a more bearable lockdown experience. Leaders were also generally more resourceful and able to access information regarding the pandemic and its latest developments. For example, many informally served as the main interlocutors between the community and the government, translating information related to the pandemic (e.g. information on masking, washing hands, social distancing measures and the use of sanitizers), and disseminated this information quickly through social media channels such as Telegram, WhatsApp and Facebook for their communities. One leader, speaking on his future wishes, stated that:

… I really want to have an event after the lockdown and MCO. And then I would like to present my community to NGOs, to locals and explain the situation, the difficulties, the challenges that we have. And ask people to understand better and I really want to appreciate a lot of people who are helping us during MCO. And understanding locals, it was really amazing. I’m happy that I’m in Malaysia … during MCO.

Similarly, local NGOs also directly impacted refugee experiences, as they were a direct source of food and cash aid for many communities during the lockdown.

They (the local NGOs) send money to us and then we buy the food from the market and distribute it. So yeah, we’re very grateful to these NGOs, if not we are supposed to live (in) the house and no food for the babies. It will be more difficult for us.

Indirectly, they also played an important role in shaping refugees’ social and institutional trust, which indirectly determine their attitudes and compliance–many refugees who had received help from local NGOs or local organizations were more willing to cooperate with orders from the government and were more likely to view local citizens and their own prospects in the country more optimistically. Although many relationships in the proposed model were interesting, particularly influential was the role of identity.

### Identity and experiences during the pandemic

In the next round of analysis, we delve deeper into one of the relationships in the model depicted in Fig 2 –specifically, the effects of identity on lived experiences, which in turn influence the level of trust among refugees. A thematic analysis [71] was conducted separately on interview transcripts from Rohingya and Syrian respondents, which was then compared and contrasted. The interviews were coded at a topic-level, similar to what was done in the first round of interviews. The full set of themes, together with the number of quotes supporting these themes, can be seen in Table 1. Certain themes were apparently only found in one group but not the other–for example, conspiracy theories, placing blame on other ethnic groups and the arrival in Malaysia by boat were only seen amongst Rohingya respondents. Ethnic Arab supremacy and the impact of the lockdowns on businesses were only seen amongst Syrian respondents. A total of 418 excerpts were obtained from the Rohingyas, and 392 from the Syrians. Taking the frequency of quotes as a measure of the “intensity” of a certain theme, there were vastly different experiences for each group on many fronts (but notably also many similarities).

We focused on five particularly striking themes for our comparison: the impact of the lockdown on jobs, repatriation, mobility, remittances and xenophobia. Many Rohingya respondents work in low-skilled jobs, such as landscaping work for local city councils (i.e. grass-cutting), garbage collection and construction. Some of these were classified as essential services, and so, many members of the Rohingya community could still work during the MCO. On the other hand, many adult Syrians, who are higher-educated and typically had previous working experience back home, lost their jobs due to the slowing economy–similar to many middle-class Malaysians. All quotes presented in this subsection come from the second round of interviews.

Those hard workers … construction worker, garbage worker, everyone is working. No problem for them. They are full-time working. Those who are teacher, so a little bit more educated, they’re facing trouble. They are not hiring. In restaurant, club, nobody is hiring them. … People like me we are facing the problem. (Rohingya respondent)

Identity also factors into discussions of repatriation. Syrians have the option to go back to Syria (and some spoke of it), but this option is not available to the Rohingya, who are stateless (although it seems that they do, in fact, want to go home).

People started even seeking help to go back home, you know. But OK, we can go back to Syria, although it is life-threatening. We could die because of bombings. Or we have to go to (forced) military service … but it’s OK. At least we don’t live in the street. So, we also started calling UNHCR to help people to go voluntarily back home, but the process is really complicated and takes a lot of time. So for example, if somebody applies to volunteer to really go back home, it would take like three months and or four months and people would have to pay the rent … and they are already without food or without money (now). (Syrian respondent)

Mobility was a theme that emerged, as movement was complicated by the MCO and the heavy presence of law enforcement officers. This issue was more severe for the Rohingyas than the Syrians, as many are illiterate. After the MCO was lifted, particularly during the time when the second-round interviews were conducted, the government had enacted a contact tracing system which generally operated in two ways: 1) all patrons of any business had to write their names and phone numbers in a physical book; or 2) scan a QR code using MySejahtera, a contact-tracing smartphone application created by the Malaysian Ministry of Health. Other contact tracing applications, such as Selangkah, also exist but are much less utilised. These contact tracing measures were problematic for the Rohingya, who did not trust the government not to reveal their contact details, were not able to read instructions to scan a QR code and were also unable to write down their own names and phone numbers when asked.

Before lockdown it was good for us, now it is not good because as uneducated guy, (to) go anywhere I need write down my name, need to take test. So I don’t know how to take this. So very difficult. (Rohingya respondent)

Remittances emerged as a fascinating difference between the two groups. The World Bank estimated that the COVID-19 crisis caused a decline in remittances by 23.1% in 2020, the “sharpest decline in recent history” [76]. Many Rohingya reported that the slashing of salaries and job losses affected their ability to send money home, which was a point of anxiety. On the other hand, Syrians seemed to be the recipients of remittances instead of senders, with money coming in from the Gulf countries and Europe.

Because it is pandemic, (we have) problems. Everyone gets half-salary. They are also surviving. Like me. If they want to survive themselves, they can’t help their family in Myanmar. If they want to help them, they cannot survive … here. (Rohingya respondent)

One more thing is that some families are here because, let’s say the husband is in Europe, so they are waiting for the transition to go to Europe or their families are in the Gulf. Yeah, so they can help them financially. (Syrian respondent)

Finally, the theme of rising xenophobia emerged for both groups, but it was evident that many Rohingyas experienced specific attacks due to negative sentiments in the media. Syrians, for example, experienced discomfort and discrimination in terms of the policy against foreigners visiting mosques–but this was a blanket ban on all non-Malaysian Muslims and not ethnicity-specific. However, Rohingyas experienced specific discrimination and hatred due to policies such as employers not allowing Rohingyas to work or telling them that their jobs were no longer available due to changing government policies after the MCO.

Right now we got reports that some employers they ask whether you are Myanmar Muslims or Rohingya. Some employers they accept those who are not Rohingya. Like non-Rohingya they accept. But especially they targeted the Rohingya. If they say they’re Rohingya, so yeah, they don’t accept the Rohingya … because of the government statement … it can harm all the Rohingya community here in Malaysia. (Rohingya respondent)

### Agents of trust and implications for policy

In our interviews, we were able to identify different “agents of trust” amongst our respondents, i.e. individuals or organizations that were pivotal in affecting a refugee’s institutional trust in the “authorities” during the MCO. Specifically, we saw that the term “authorities” was a very complex construct for refugees. For example, when refugees spoke about the “government”, they typically separated this construct into five distinct categories: healthcare workers, civil servants, politicians, immigration officers and the police, as illustrated by three quotes below about immigration officers, healthcare workers and politicians respectively.

During early MCO, the government announced that they won’t arrest for immigration reason … those who don’t have UNHCR (cards), don’t have visa, (can still) go to the hospital and report about the (COVID) case. But now if something like that happens I will be extremely surprised.

… they said they get scared at some point when the (immigration and police) authorities start screaming you know. In Malay. Some of our community members they don’t really get the language so maybe they don’t know what the instruction is about. So, they don’t come out from the house and the (immigration and police) officers get angry. Those things actually happen. But not really in a bad way, so we never get reports like anyone is … physically abused. Everything goes well and especially, people are in touch most of the time with the medical team. Government medical team … the way they treat (refugees), according to everyone in that (affected) area … is a lot flexible and polite you know. Instead of the government enforcement who can be very impatient. So, we don’t do anything that is … drawing the anger or attention of them.

The government is very new … in the government there are political parties, they have their own problems right now. So, (local) people are busy, and politicians are busy with their own problems.

Other agents of trust and their categories are as follows: home (generally and family), Malaysians (locals, employers and NGOs), the UNHCR and social networks (refugee community, community leaders and leaders from other refugee communities).

This very clear demarcation in the minds of our respondents has implications for policies, whereby specific entities operating within a country do have the power to win the trust of refugee communities, and can consequently influence their attitudes towards and compliance with public health measures. For example, although the Ministry of Health could have been lumped in with the broader Malaysian government, many respondents reported trusting doctors and nurses significantly more than politicians. This implies that healthcare workers, perhaps through general education campaigns, can in fact reach the refugee community in ways that politicians, immigration authorities or the police cannot. We suggest that these agents of trust are identified by organizations or governments interested in engaging with the refugee community, and channeling resources to these agents to encourage compliance with and obedience of actions that have strong externalities, such as mask-wearing, testing, and social distancing.

The second policy discussion pertains to the process model in Fig 2. Refugee community leaders are often engaged by local NGOs for the distribution of aid to their respective communities. Refugee leaders are pivotal in many situations, typically serving as interlocutors between their own community and local society. However, from our interviews, it is also clear that each leader tends to serve only a specific portion of the overall refugee community–for example, a Rohingya community leader living in the state of Selangor probably only serves Rohingya individuals and families within a limited geographical area. Repeatedly relying on the same community leader for the distribution of aid outside that area, such as food or cash, may indicate that large portions of the broader community are left out. Although it is often more convenient for an NGO or local government to work with the same refugee leader each time aid needs to be distributed, this may be problematic–many of our respondents from the second round of interviews reported that they either did not know a refugee leader (and by extension had no access to external help from local NGOs) or were not aware that aid could be obtained through the leader, suggesting that proximity largely determined whether one received aid or not.

It is also very difficult, because many of NGO(s) are based in KL. And the refugee community is everywhere in Malaysia, including Sabah and Sarawak (Malaysia Borneo) as well. We can’t look after everyone. So, we have very limited reach … that’s the thing.

I have been trying to get help from NGOs and also from UNHCR. Normally I don’t know any NGO. Still I’d been trying. I haven’t got any financial help from any others.

This brings us to our second policy recommendation: organizations or governments involved in aid distribution should maximize the set of community leaders whom they work with, potentially also sequentially rotating between different leaders if resources are limited. This will help ensure maximum coverage in the distribution of aid.

Thirdly, the experiences of refugees during the COVID-19 pandemic stem from the systemic vulnerabilities caused by the absence of domestic governing and regulatory mechanism pertaining to refuge populations in Malaysia. Without legal recognition, refugees are denied basic rights including the rights to employment and access to affordable public services including healthcare and education. The refugees also have no rights to safety and protection in Malaysia as they are deemed illegal migrants under the [77]. As the result of the marginalisation, most of the refugees are living in poverty and precariousness coupled with constant fear of arrest by the authorities. They are also exposed to higher risk of infections due to their living conditions and the fear of seeking healthcare services when needed. Their vulnerabilities are exacerbated during the pandemic as illustrated above. Refugees have to cope with the consequences of the public health crisis and economic shock following the containment measures without any support from the government’s policy responses towards the pandemic. The increase in immigration raids has also eroded their institutional trust. While humanitarian and civil society organisations have stepped in to fill the gap in assisting the refugees, it is only a band-aid solution that does not last. A more sustainable approach would be providing legal recognition to refugees particularly in terms of temporary rights to employment. When asked about what is the most urgent during the pandemic, one of the participants remarked,

The most urgent need will be like the‥ the job la. If we get job, then we will be able to pay for our rental and for the food. I think should be OK. We like to get‥ to get work. And get job. If we can get job, I mean all the problems can be solved la‥ 75% I can say.

## Conclusion

COVID-19 does not discriminate, but it does disproportionately increase the vulnerability of marginalized populations. This study documents and maps the multifaceted experiences among refugee populations during this pandemic. In addition to heightened risks of infection, this study reveals the plight of refugee populations that have been facing social and economic insecurity prior to the pandemic. The loss of livelihoods following the MCO generated negative ripple effects in terms of their access to basic necessities like food and shelter. At the same time, they also face disproportionate vulnerability due to their lack of rights, which excludes them from full access to public health facilities as well as economic stimulus packages that aim to cushion the resulting negative economic shocks. Illiteracy and lack of linguistically accessible information have restricted them from obtaining vital information for responding to this public health emergency. The situation is exacerbated by the rise of xenophobic policies and behaviors, which cause severe anxiety among the community and threaten their sense of security. While NGOs and community leaders have emerged to fill the void left by the government (itself seen as a complex construct by refugees) by delivering support and aid to the refugee community, the paucity of community-led assistance, which is concentrated in certain areas, may leave out others who are under the radar.

A conceptual model was constructed based on the findings from the first round of interviews. The proposed framework establishes a link between policy changes and trust in domestic authority figures, mediated by the multifaceted experiences of refugee populations. In line with the literature (42,49,50), we found that there are two types of trust among refugees: social trust and institutional trust. The level of trust, in turn, influences the attitudes towards and compliance with public health measures in containing the spread of the virus among refugee populations. The conceptual model is validated through a follow-up comparative study between Rohingya and Syrian refugees which shows that refugees of different identities do face different sets of challenges during the pandemic, which in turn determines their levels of trust and compliance. Besides contributing to the existing literature on trust, these findings fill the gap in the literature pertaining to how refugee populations experience the pandemic and lockdowns in Malaysia, which may be extrapolated to refugee experiences in other developing countries. From a policy perspective, this study contributes to a better understanding of refugee vulnerability, which is important in formulating a holistic response to COVID-19. This study also calls for an inclusive policy that focuses on building trust with the refugee community in order to contain the pandemic effectively.

## Supporting information

S1 File (PDF)
