# Supplementary material for: Identity, trust, and the experiences of refugees during a COVID-19 lockdown
Source: PLoS One. 2022 Aug 1;17(8):e0271977. doi: 10.1371/journal.pone.0271977 (PMC9342751; doi:10.1371/journal.pone.0271977)
Supplement: S1 File — (PDF) [file pone.0271977.s001.pdf]

ASIA School of Business

in collaboration with MIT Sloan Management

STUDY: HOW HAS THE PANDEMIC AFFECTED THE REFUGEE COMMUNITY IN MALAYSIA?

Prof. Melati Nungsari, Prof. Sam Flanders, Chuah Hui Yin

Explanation of the study and consent:

My name is _________ and I'm a researcher with the Asia School of Business. We are conducting a study on the impact of the COVID crisis on the refugee community. As researchers, we uphold strict academic and ethical standards when conducting studies with human participants. Before we start, I need to inform you on the following points and get your consent on the following:

- The interview will be conducted in either Bahasa Melayu or English, or both, according to your preference.
- We are not collecting any personal data and your answers are all confidential. We will never share any identifying data collected from you today with anybody, including the government. Anything reported in any publication from here onwards using the data collected in this study will be in a form that will not be able to be traced back to you.
- The interview will be recorded but we will keep your identity anonymized. The recording will be stored online at a secure, private location and will be completely deleted as soon as the interview has been transcribed.
- You may at any time choose to withdraw from this research without giving any reason.
- You will receive RM20 as a small token of appreciation for your time at the end of the interview.

If you agree to these terms and understand everything that we have told you, please mark X or use your first initial here.

Background:

- Can you tell me about yourself?
  - Prompt: How long have you been in Malaysia; Who do you stay with?
- What is the most significant change/biggest impact you face because of the pandemic or the lockdown?

Economic:

- Did you or any of your friends became unemployed since COVID-19 started?
  - If yes:
    - Where did you/they work before the pandemic and what did you/they work as?
    - How have you/they been coping in the last 2-3 months?
    - Have you/they pursued any other ways to generating income?
- Do you or any members in your community that run businesses?
  - If yes:
    - How has COVID-19 affected these businesses

- Do you think job opportunities and work for people in your community will return after the lockdown is eased?

Health

- Now that COVID-19 is ongoing, are you or your friends still getting medical treatment when they are sick?
- Do you know of anybody in your community who has COVID-19, or that you suspect has COVID-19? Or do you have the experience of infected COVID-19?
  - If yes:
    - What happened to you/them? Did you/they seek medical treatment?
- Do you and people in your community feel safe to go to the hospital or clinic to get medical treatment for non COVID-19 illnesses?
- Do you and people in your community feel safe to go to the hospital if they suspect they have COVID-19?
- Do you understand what contact tracing is?
  - If no, (interviewer) explain about contact tracing.
    - If you or people in your community are approached by an official from the government who is doing contact tracing, will you be comfortable revealing the names of people in your community who might have had encounters with infected people?

Social and Security

- In this time of COVID-19, how do you think Malaysians feel now towards refugees?
- How have these feelings/sentiments from Malaysians affected your and your community's day-to-day life?
- Were you or any members of your community get arrested during the recent immigration raids?
  - If yes:
    - How many of them?
    - What happened to them after they were arrested?
- How are the children in your community coping with the pandemic?
- Do the children in your community still go to school or learn at home?
- How are the women in your community coping with the pandemic?

Overall

- What are your and your community's most urgent needs right now?
- Have any organizations been helping or aiding you in any of these needs?
  - If yes, who?
- How do you and your community share information about COVID-19?
- What do you think is the most effective channel to disseminate information to your community?
- Who would you approach first for assistance or support?
- Is there anything else you would like us to know about you and your community?

STUDY: HOW HAS THE PANDEMIC AFFECTED THE REFUGEE COMMUNITY IN MALAYSIA?

Prof. Melati Nungsari, Prof. Sam Flanders, Chuah Hui Yin

Explanation of the study and consent:

My name is _________ and I'm a researcher with the Asia School of Business. We are conducting a study on the impact of the COVID crisis on the refugee community. As researchers, we uphold strict academic and ethical standards when conducting studies with human participants. Before we start, I need to inform you on the following points and get your consent on the following:

- The interview will be conducted in either Bahasa Melayu or English, or both, according to your preference.
- We are not collecting any personal data and your answers are all confidential. We will never share any identifying data collected from you today with anybody, including the government. Anything reported in any publication from here onwards using the data collected in this study will be in a form that will not be able to be traced back to you.
- The interview will be recorded but we will keep your identity anonymized. The recording will be stored online at a secure, private location and will be completely deleted as soon as the interview has been transcribed.
- You may at any time choose to withdraw from this research without giving any reason.
- You will receive RM20 as a small token of appreciation for your time at the end of the interview.

If you agree to these terms and understand everything that we have told you, please mark X or use your first initial here.

Background:

- What community do you represent?
- How long have you been in Malaysia?
- How many individuals are in your community?
- Can you please describe your community? *(For interviewer: If possible, focus on general descriptions on age distribution, gender distribution, and occupations)*
- What is the most significant change/biggest impact the community face because of the pandemic or the lockdown?

Economic:

- Have any members in your community become unemployed since COVID-19 started?
    - If yes:
        - Where did they work before the pandemic and what did they work as?
        - How have they been coping in the last 2-3 months?
        - Have they pursued any other ways to generating income?
- Do you know of any members in your community that run businesses/social businesses?

    - If yes:
        - How has COVID-19 affected these businesses
- As a community leader, do you think job opportunities and work for people in your community will return during PKPP?

Health

- Now that COVID-19 is ongoing, are people in your community still getting medical treatment when they are sick?
- Do you know of anybody in your community who has COVID-19, or that you suspect has COVID-19?
    - If yes:
        - What happened to them? Did they seek medical treatment?
- Do you and people in your community feel safe to go to the hospital or clinic to get medical treatment for non COVID-19 illnesses?
- Do you and people in your community feel safe to go to the hospital if they suspect they have COVID-19?
- Do you understand what contact tracing is?
    - If no, explain contact tracing.
    - If yes:
        - If you or people in your community are approached by an official from the government who is doing contact tracing, will you be comfortable revealing the names of people in your community who might have had encounters with infected people?

Social and Security

- In this time of COVID-19, how do you think Malaysians feel now towards refugees?
- How have these feelings/sentiments from Malaysians affected your and your community's day-to-day life?
- Were there any members of your community who get arrested during the recent immigration raids?
    - If yes:
        - How many of them?
        - What happened to them after they were arrested?
- How are the children in your community coping with the pandemic?
- Do the children in your community still go to school or learn at home?
- How are the women in your community coping with the pandemic?

Overall

- What are your and your community's most urgent needs right now?
- Have any organizations been helping or aiding you in any of these needs?
    - If yes, who?
- How do you and your community share information about COVID-19?
- What do you think is the most effective channel to disseminate information to your community?
- Who would you approach first for assistance or support?
- Is there anything else you would like us to know about you and your community?
